# Use of a novel “Split” ventilation system in bench and porcine modeling of acute respiratory distress syndrome

**DOI:** 10.14814/phy2.15452

**Published:** 2022-09-09

**Authors:** Pierce Geoghegan, Jennifer Clarke, Grace Hogan, Aoife Keogh, Hannah Marsh, Karen Donnelly, Natalie McEvoy, Aoife Doolan, Stephen F. Madden, Ignacio Martin‐Loeches, Michael Power, John G. Laffey, Gerard F. Curley

**Affiliations:** ^1^ Department of Anaesthesia and Critical Care Royal College of Surgeons in Ireland Dublin Ireland; ^2^ Beaumont Hospital Dublin Ireland; ^3^ Tallaght Hospital Dublin Ireland; ^4^ Data Science Centre Royal College of Surgeons in Ireland Dublin Ireland; ^5^ Department of Intensive Care St. James Hospital Dublin Ireland; ^6^ Department of Anaesthesia and Critical Care Galway University Hospital Galway Ireland

**Keywords:** acute lung injury, acute respiratory distress syndrome, lung protective ventilation, split ventilation

## Abstract

Split ventilation (using a single ventilator to ventilate multiple patients) is technically feasible. However, connecting two patients with acute respiratory distress syndrome (ARDS) and differing lung mechanics to a single ventilator is concerning. This study aimed to: (1) determine functionality of a split ventilation system in benchtop tests, (2) determine whether standard ventilation would be superior to split ventilation in a porcine model of ARDS and (3) assess usability of a split ventilation system with minimal specific training. The functionality of a split ventilation system was assessed using test lungs. The usability of the system was assessed in simulated clinical scenarios. The feasibility of the system to provide modified lung protective ventilation was assessed in a porcine model of ARDS (*n* = 30). In bench testing a split ventilation system independently ventilated two test lungs under conditions of varying compliance and resistance. In usability tests, a high proportion of naïve operators could assemble and use the system. In the porcine model, modified lung protective ventilation was feasible with split ventilation and produced similar respiratory mechanics, gas exchange and biomarkers of lung injury when compared to standard ventilation. Split ventilation can provide some elements of lung protective ventilation and is feasible in bench testing and an in vivo model of ARDS.

## Graphical abstract

Split ventilation (using a single ventilator to ventilate multiple patients) is technically feasible. However, connecting two patients with acute respiratory distress syndrome (ARDS) and differing lung mechanics to a single ventilator is concerning. In this study we demonstrate that split ventilation can provide some elements of lung protective ventilation and is feasible in bench testing and an in vivo model of ARDS.

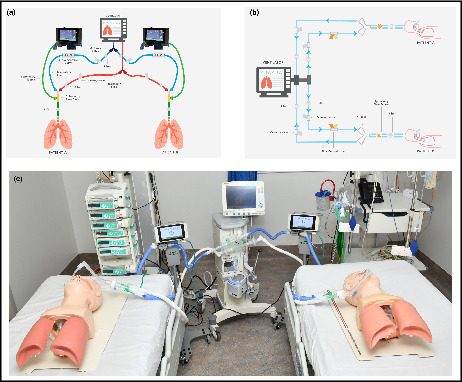

## 1 INTRODUCTION

The COVID‐19 pandemic has been characterized by large numbers of patients developing respiratory failure (Ranney et al., 2020). Concerns have been expressed about the ability to invasively ventilate large numbers of patients presenting nearly simultaneously to individual hospitals with severe respiratory failure. “Rationing” of ventilators is a feared scenario and, tragically, has already occurred (Rosenbaum, 2020). “Split” ventilation (using a single ventilator to ventilate multiple patients) is a potential solution but the safety of this strategy is controversial (Branson & Rubinson, 2006; Cherry et al., 2020; Cook, 2020; Herrmann et al., 2020; Laffey et al., 2020; Mancebo et al., 2020).

Prior to the COVID‐19 pandemic, “split” ventilation had shown promising results in some bench (Neyman & Irvin, 2006) and modest in vivo experiments (Paladino et al., 2008; Smith & Brown, 2009). Enthusiasm for the idea was tempered by safety concerns around ventilatory independence (Branson et al., 2012). Nevertheless, there is at least one well known example of successful use of split ventilation in a mass casualty situation (Menes & Plaster, 2017).

COVID‐19 renewed interest in the topic and several bench studies demonstrated feasibility of “split” ventilation of test lungs (Bishawi et al., 2020; Boyer et al., 2020; Clarke et al., 2020; Han et al., 2020; Srinivasan et al., 2020; Tonetti et al., 2020). Limited experiments have been performed in animals without lung injury (Stiers et al., 2020) and case series exist of “split” ventilation in COVID‐19 patients (Beitler et al., 2020; Levin et al., 2020). These data have not reassured field experts (Cook, 2020; Laffey et al., 2020; Mancebo et al., 2020) and safety concerns dominate relevant consensus statements (The Society of Critical Care Medicine (SCCM) AAfRCA, American Society of Anesthesiologists (ASA), Anesthesia Patient Safety Foundation (APSF), American Association of Critical‐Care Nurses (AACN), and American College of Chest Physicians (CHEST), 2020).

There is an absence of in vivo experimentation comparing “split” ventilation to standard or “single” ventilation in animal models of acute respiratory distress syndrome (ARDS). We hypothesized that, in a porcine model of ARDS, standard ventilation would be superior to “split” ventilation across a range of markers of respiratory mechanics, gas exchange, and biological markers of lung injury.

## 2 METHODS

### 2.1 Ethical approval

The study received approval from the relevant licensing body, the Health Products Regulatory Authority of Ireland (HPRA, Dublin, Ireland).

### 2.2 Split ventilation system

**FIGURE 1:**
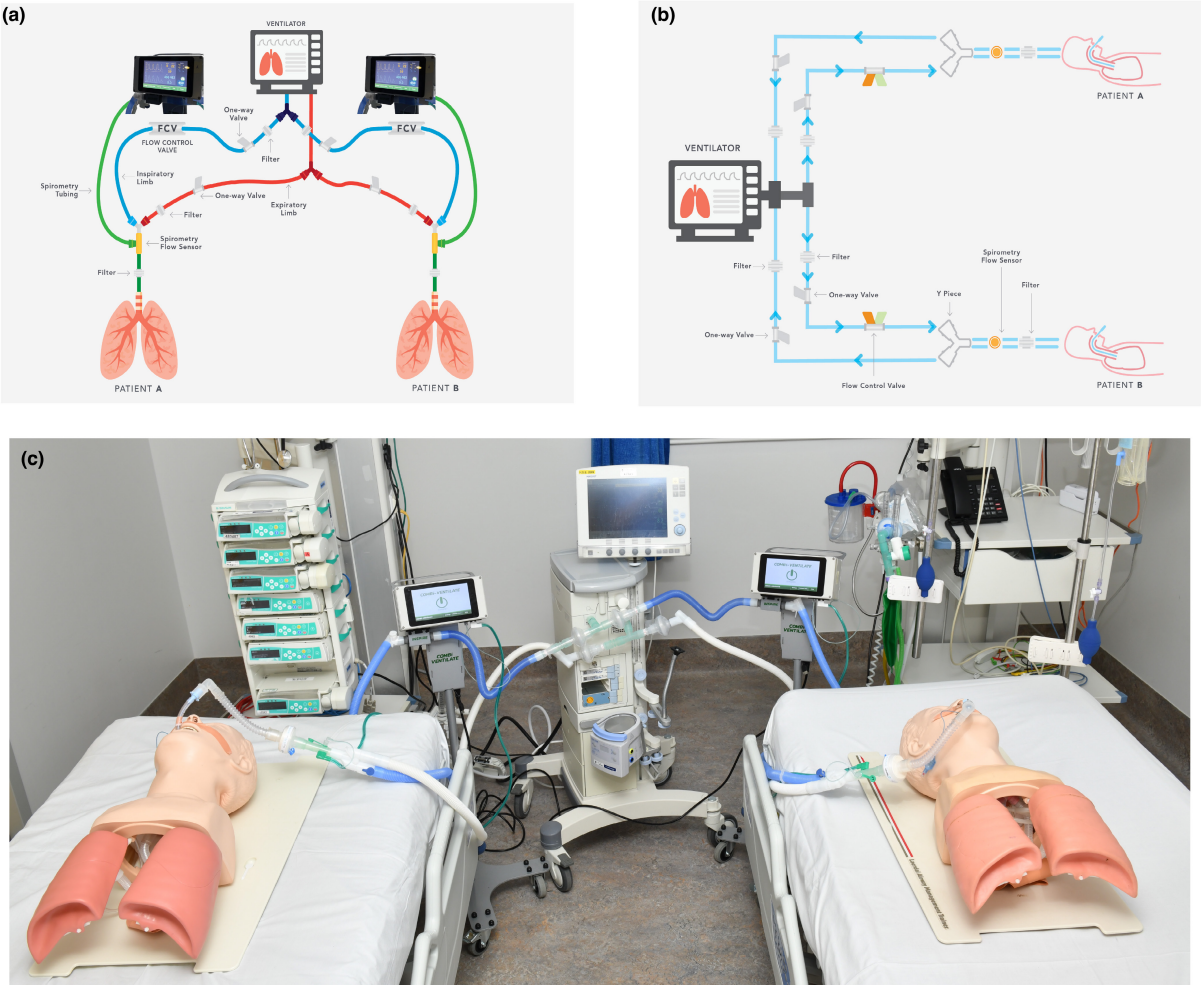
Design of the Combi‐Ventilate “Split” ventilation system (a) Schematic of Combi‐Ventilate circuit setup. (b) Circuit diagram of Combi‐Ventilate system. (c) Photograph of Combi‐Ventilate system connected to a Servo‐I ventilator (Maquet, Germany) and two test lungs.

For the purposes of our experiments, we utilized a novel investigational split ventilation system called the Combi‐Ventilate system (Combilift, Ireland). A schematic of the system is shown in Figure 1. The system setup is described in detail in the online digital supplement (S1.1 and Table S6). Briefly, it uses valves and flow regulators in parallel limbs to independently control tidal volume for two patients connected to a “parent” ventilator operating in a “pressure control” ventilation mode. It incorporates an electronically controlled flow regulating valve and touch‐screen display. Users can set tidal volume, visualize pressure, flow and volume waveforms for each patient and set alarm limits i.e., users can individually titrate ventilation.

### 2.3 Benchtop testing

Compatibility of the split ventilation system with the Servo‐I ventilator (Maquet) was assessed by ventilating two Michigan test lungs (Model 2600i, Michigan Instruments, Grand Rapids) as schematised in Figure 1. Similar testing was performed with additional ventilators (V800/Evita 4, Dräger Medical) to ensure generalisability (online digital supplement, Tables S2–S5).

Independence of ventilation was tested by:

1. assessing delivery of discrepant tidal volumes at matched test lung compliance (80 ml/cmH_2_O;50 ml/cmH_2_O;30 ml/cmH_2_O).
2. confirming that altered compliance/resistance in one test lung did not affect ventilation of the other test lung.

### 2.4 Feasibility of “Split” ventilation in a porcine model of ARDS

#### 2.4.1 Animal preparation, treatment groups, and mechanical ventilation

Thirty community‐bred female Landrace pigs (mean 40 kg ± 10 kg) were anesthetized, intubated, mechanically ventilated, monitored and haemodynamically supported per protocol. All experiments were conducted in compliance with the ARRIVE guidelines and in line with the directive 2010/63/EU as transposed into Irish law by SI No 543 of 2012. All procedures were approved by the relevant licensing body, the Health Products Regulatory Authority of Ireland (HPRA).

Replicates were assigned to one of four treatment groups—“single” ventilated uninjured (*n* = 5), “single” ventilated injured (*n* = 5), “split” ventilated uninjured (*n* = 10) and “split” ventilated injured (*n* = 10), with five replicates per group and block allocation of animals in pairs. “Single” ventilation was with a standard ventilator (Evita 4; Dräger Medical) while “split” ventilation used the Combi‐Ventilate system.

All animals were ventilated in a “volume‐control” mode for 6.5 h targeting plateau pressure ≤30 cmH_2_O, tidal volume ≤8 ml/kg body weight, pH ≥ 7.15, and PaO_2_ ≥ 8 kPa or SaO_2_ ≥ 88%. In “single” ventilated animals, PEEP was set according to the ARDSnet “low” PEEP protocol (Acute Respiratory Distress Syndrome Network et al., 2000). In “split” ventilated animals shared PEEP was titrated to the lowest value permitting oxygenation targets as outlined above. No animals were excluded in the analysis.

Detailed methods are contained in the online digital supplement (S1.2).

#### 2.4.2 Lung injury protocol

Lung injury was accomplished by endobronchial administration of acid. HCl 0.05 N, pH 1.41, was prepared and instilled (8 ml/kg body weight) at the right cranial lobe bronchus, the right main bronchus, and the left main bronchus, in the ratio of 1:2:3 over 3 min by means of a flexible bronchoscope (Ambu®ascope™). We instilled the acid directly after intubation and allowed 60 min post instillation for lung injury to become established. In uninjured animals, bronchoscopy was performed at identical timepoints, but without any instillation of acid or vehicle.

### 2.5 Measurements

#### 2.5.1 Respiratory mechanics and gas exchange

Total respiratory system compliance, plateau pressure, PEEP and gas exchange were measured regularly. Total respiratory system compliance was calculated as tidal volume/(plateau pressure‐PEEP). For “single” ventilated animals, plateau pressure was measured during an end inspiratory hold and PEEP during an end expiratory hold. For “split” ventilated animals, plateau pressure was measured by the Combi‐Ventilate spirometer during an end inspiratory hold on the “parent” ventilator. Similarly, PEEP was measured by the Combi‐Ventilate spirometer system during an end expiratory hold maneuver on the “parent” ventilator (Combi‐Ventilate measurements of PEEP and plateau pressure were validated separately, Table S7).

#### 2.5.2 Bronchoalveolar lavage fluid sampling for protein quantification and cytokine analysis

ELISA kits from R&D systems were used to measure the levels of of IL‐6 (DY686), IL‐10 (DY693B) and TNFα (DY690B) in bronchoalveolar lavage fluid (BALF) immediately before injury, post‐injury and 6 h later. Similarly, total protein levels in BALF were quantified using the Pierce BCA protein assay kit (#23227) from Thermo Scientific. Detailed methods can be found in the online data supplement.

#### 2.5.3 Wet‐to‐dry ratio

Animals were euthanised at the end of the experiment and two tissue samples from the caudal lobe of each lung were taken, weighed, dried for 72 hours at 60°C, weighed again and the wet‐to‐dry ratio calculated.

#### 2.5.4 Usability assessment

To assess the usability of the “Combi‐Ventilate” device, a convenience sample of critical care medical staff (*n* = 20) operated the device in simulated conditions after minimal specific training (a single 20‐min video tutorial). Operators had to (1) correctly assemble the system, (2) set “parent” ventilator and Combi‐Ventilate module parameters to deliver a predetermined tidal volume to both test lungs, and (3) respond correctly to simulations of abrupt changes in compliance. Participants were given unrestricted access to an instruction manual, but no other assistance was permitted. Participants were deemed either successful or unsuccessful in each of the 3 domains by two independent observers.

#### 2.5.5 Sample size and statistical analysis

For the porcine model, no similar or pilot data were available for formal power analysis and instead sample size was optimized using the resource equation method (Festing & Altman, 2002; Mead, 1988). In the main analysis a Shapiro–Wilks test was used to test for normality for each metric. Subsequently, a two‐way repeated measures ANOVA was used to determine if there were significant differences between ventilation type and injury status and if this varied over time. Where significant differences occurred, post‐hoc testing of “single” ventilated injured versus uninjured and “split” ventilated injured versus uninjured were performed using a *t*‐test and *p*‐values adjusted using the Benjamini–Hochberg method (Benjamini & Hochberg, 1995). Analysis was conducted in R (R Development Core Team, 2010).

## 3 RESULTS

### 3.1 Benchtop testing

Real patients require different tidal volumes. Delivery of discrepant tidal volumes was assessed over a range of matched compliance (online digital supplement, Table S1). At matched compliance of 80 ml/cmH_2_O and driving pressure of 20cmH_2_O, tidal volumes could differ by a ratio of 4.4:1. Even at low matched compliance (20 ml/cmH_2_O), tidal volumes were as discrepant as 2.5:1. Therefore, patients with significantly different predicted body weights could be accommodated on the same split ventilation system.

**FIGURE 2:**
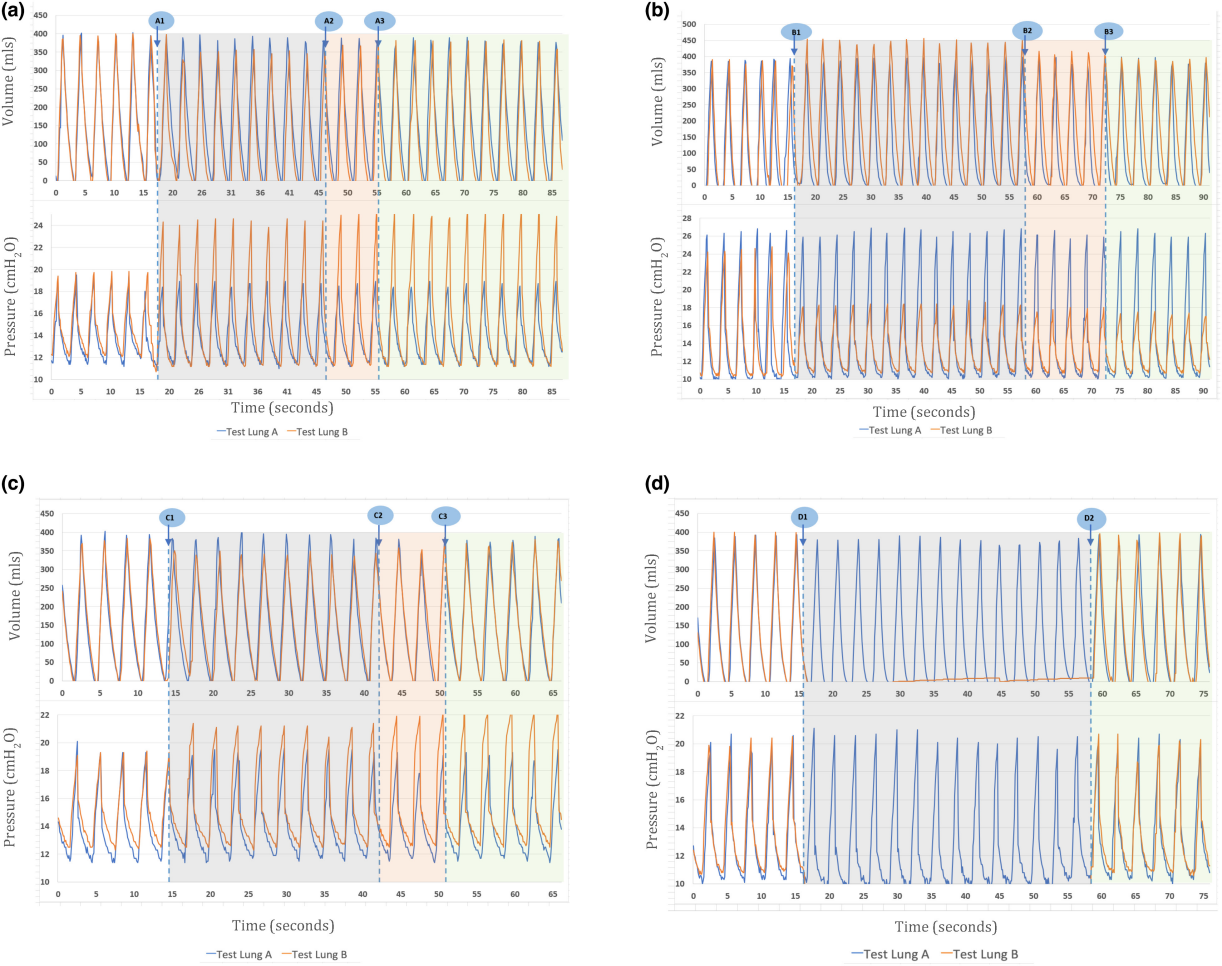
Volume and pressure waveforms of two test lungs undergoing “Split” ventilation. (a) The effect of a decrease in compliance in test lung B on gas flow and respiratory mechanics in test lungs A and B. Marker A1 represents the point that compliance decreased in test lung B. As expected, tidal volumes decrease in test lung B but no significant change in tidal volumes is seen in test lung A. Marker A2 is the point at which the tidal volume retargeting algorithm is initialized by the user. Marker A3 is the point at which original tidal volume is restored. (b) The effect of an increase in compliance in test lung B on gas flow and respiratory mechanics in both test lungs. Marker B1 represents the point at which compliance increases in test lung B, with a corresponding reduction in airway pressure and increase in tidal volumes in test lung B. Test lung A is not significantly affected by this. Marker B2 is the point at which the tidal volume retargeting algorithm is initialized by the user. Marker B3 is the point that the original volume is restored to test lung B. (c) The effect of an increase in resistance in test lung B on gas flow and respiratory mechanics in both test lungs. Resistance in test lung B increases at marker C1, with corresponding increased airway pressure and decreased tidal volume in that test lung but without any significant changes in test lung A. Marker C2 is the point at which the tidal volume retargeting algorithm is initialized by the user. Marker C3 is the point that the original tidal volume is restored. (d) The effect of a disconnection in test lung B. Test lung B is disconnected at the time point represented by marker D1. There is no significant change in gas flow or respiratory mechanics in test lung A and original conditions are restored upon reconnection of test lung B (Marker D2).

A concern in split ventilation is that altered respiratory mechanics in one patient will negatively impact the second patient's ventilation. Therefore, volume and pressure waveforms in two test lungs during simulations of abrupt changes in respiratory mechanics in one test lung were examined to confirm this would not occur in the “split” ventilation system we used (Figure 2). Figure 2a shows the effect of reduced compliance in test lung B on gas flow and respiratory mechanics in test lungs A and B. As expected, tidal volumes drop in test lung B, but are unchanged in test lung A. Following user activation of the electronically controlled flow regulating valve for test lung B, tidal volumes are automatically restored to test lung B within 10 seconds. Tidal volumes in test lung A are unaffected.

We also observed ventilatory independence during an increase in compliance in test lung B (Figure 2b). A similar pattern was observed on manipulation of resistance in test lung B (Figure 2c). Despite reduced tidal volumes and increased airway pressures in test lung B on increasing resistance in test lung B, gas flow and airway pressure were unaffected in test lung A. Even during a disconnect of test lung B, ventilation to test lung A was unchanged. A. Overall, this demonstrates that neither altered compliance/resistance of a single test lung, nor subsequent adjustment of the flow regulating valve, meaningfully affect the ventilation of the partner test lung.

### 3.2 Feasibility of “Split” ventilation in a porcine model of ARDS

#### 3.2.1 Respiratory mechanics and gas exchange

**FIGURE 3:**
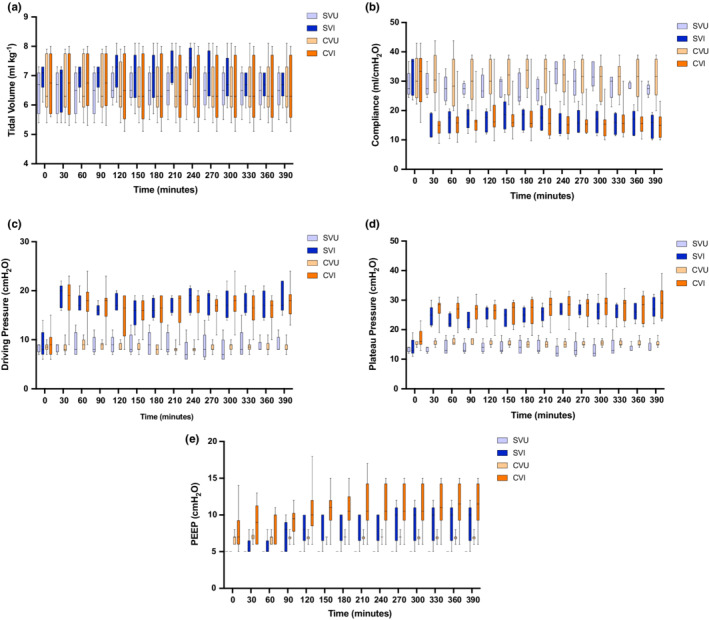
Respiratory mechanics of injured and uninjured animals undergoing “Split”(Combi‐Ventilate) or “Standard” ventilation. Panel (a) shows tidal volume (ml/kg) over time. There were no significant differences between groups receiving “split” compared to standard ventilation (*p* = 0.289). Panel (b) displays compliance measurements over time in all groups. Post‐injury, injured animals had reduced levels of compliance compared to uninjured animals (*p* < 0.001). Panel (c) shows driving pressure over time. Post‐injury, driving pressures were higher in injured vs uninjured animals (*p* < 0.001). Panel (d) shows plateau pressures measured over time. Plateau pressures were higher in injured animals versus uninjured (*p* < 0.001). Panel (e) demonstrates positive end‐expiratory pressure (PEEP) in all groups over time. PEEP was higher in shared ventilated animals in both uninjured and injured groups compared to those undergoing standard ventilation (*p* = 0.012). For panels (a–c), there was no significant difference between animals who received “split” ventilation compared to standard ventilation. For panel (d), there were no significant differences between standard and split ventilated animals after 30 min. For panel (e) only, there were significant differences between shared and standard ventilation in uninjured animals at every time point (all adjusted *p* < 0.05). Data are presented as box and whisker plots. The box indicates the interquartile range and contains a line at the median value. The whiskers denote the range. Differences in treatments were determined using a two‐way repeated measures ANOVA. Pairwise comparisons between ventilation types were performed using a *t*‐test and *p*‐values were adjusted using the Benjamini–Hochberg method. SVU = Single (“Standard”) Ventilator Uninjured (*n* = 5); SVI = single (“Standard”) ventilator injured (*n* = 5); CVU = combi‐ventilate(“Split”) uninjured (*n* = 10); CVI = combi‐ventilate (“Split”) injured (*n* = 10).

Adherence to “lung protective” ventilation in “split” and “single” ventilated animals was compared in a porcine model of ARDS (*n* = 30). Tidal volumes were maintained in a “lung protective” range (≤8 ml/kg body weight) throughout (Figure 3a) and did not differ significantly between “single” and “split” ventilation (*p* = 0.289). This indicates feasibility of low tidal volume ventilation, even in injured animals.

Figure 3b demonstrates significantly lower post‐injury total respiratory system compliance in injured animals (*p* < 0.001). Because ventilator‐induced lung injury is associated with reduced compliance (Ricard et al., 2003), additional ventilator‐induced injury from split ventilation should be associated with reduced compliance. However, there were no significant differences in compliance between “single” and “split” ventilated animals (all adjusted *p* > 0.05). Therefore, compliance measurements produced no evidence to support additional ventilator‐induced lung injury attributable to split ventilation.

Figure 3c,d demonstrate that driving and plateau pressures were significantly higher in injured compared to uninjured animals (*p* < 0.001 for both comparisons). Since plateau and driving pressures are key surrogates of barotrauma (Ricard et al., 2003), they were compared in “single” and “split” ventilated animals but no significant differences were observed (all adjusted *p* > 0.05 for driving pressure throughout, and for plateau pressure beyond 30 min). Therefore, standard ventilation did not appear superior to “split” ventilation with respect to these surrogates of barotrauma.

Because PEEP was not individualized in the test system, PEEP should be higher in “split” compared to “single” ventilated animals. Indeed, this was the case (Figure 3e). PEEP was significantly higher in “split” compared to “single” ventilated animals (*p* = 0.012). This was most consistent in uninjured animals (all adjusted *p* < 0.05).

**FIGURE 4:**
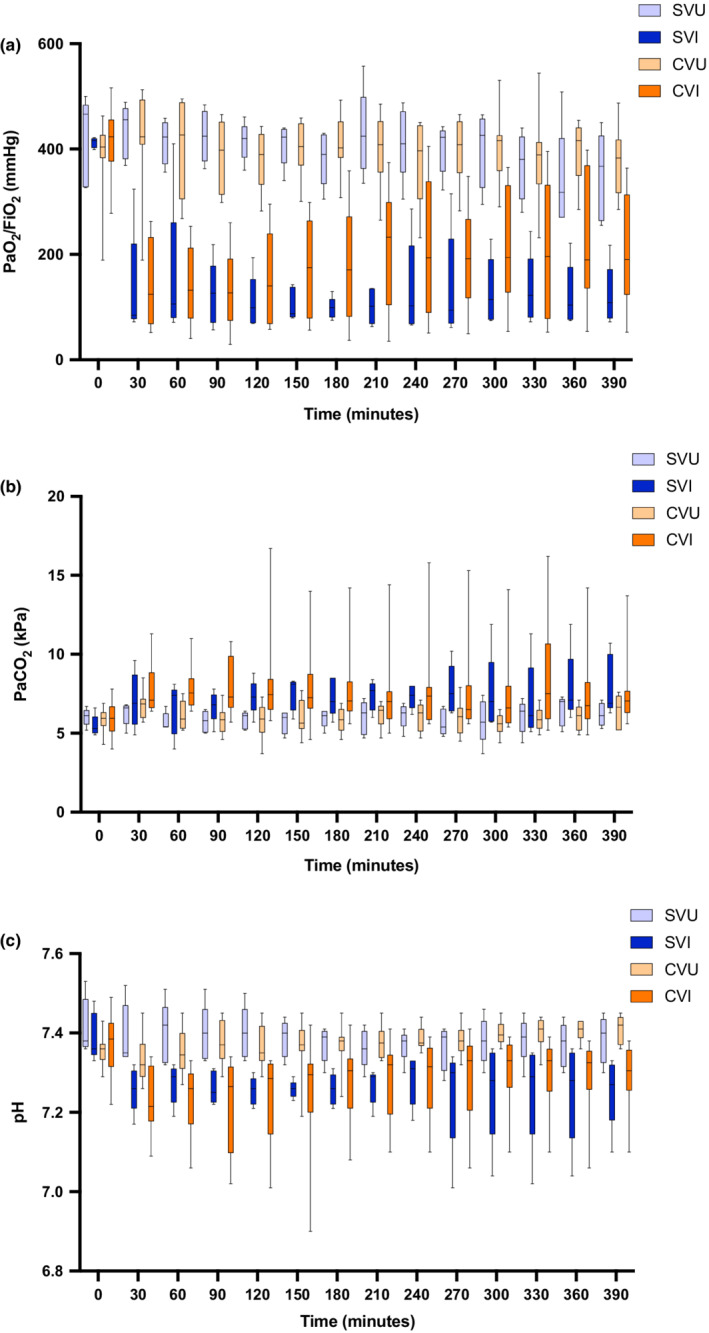
Measurements of gas exchange in “Split”(Combi‐Ventilate) and “Standard” ventilation groups of injured and uninjured animals. Panel (a) demonstrates PaO_2_/FiO_2_ (P:F) ratio measurements in all groups over time. Post‐injury, animals in both the “split” and standard ventilation injured groups had lower P:F ratios compared to uninjured animals (*p* < 0.001). Panel (b) displays PaCO_2_ levels in all groups over time. Higher PaCO_2_ levels were recorded in injured animals compared to uninjured animals (*p* = 0.014). Panel (c) demonstrates pH measurements in all groups over time. Uninjured animals had a higher pH compared to injured animals (*p* = 0.005) and there was no significant difference between “split” or standard ventilation animals. For panels (a–c), there was no significant difference between animals who received “split” ventilation compared to standard ventilation. Data are presented as box and whisker plots. The box indicates the interquartile range and contains a line at the median value. The whiskers denote the range. Differences in treatments were determined using a two‐way repeated measures ANOVA. Pairwise comparisons between ventilation types were performed using a t‐test and p‐values were adjusted using the Benjamini‐Hochberg method. SVU = Single (“Standard”) Ventilator Uninjured (*n* = 5). SVI = Single (“Standard”) Ventilator Injured (*n* = 5); CVU = Combi‐Ventilate (“Split”) Uninjured (*n* = 10); CVI = Combi‐Ventilate (“Split”) Injured (*n* = 10).

Facilitating gas exchange is a key function of mechanical ventilation systems, therefore gas exchange parameters were compared across groups (Figure 4). PF ratios were significantly lower in injured animals (Figure 4a, *p* < 0.001). There were no significant differences in PF ratio between “single” and “split” ventilated animals (all adjusted *p* > 0.05). Therefore, standard ventilation did not appear superior to “split” ventilation in facilitating oxygenation.

Similarly, if “split” ventilation permits individualized ventilation, PaCO_2_ and pH levels should not vary between “split” and “single” ventilation. Consistent with this, while PaCO_2_ was higher in injured versus uninjured animals (*p* = 0.014), there were no significant differences between “single” and “split” ventilated groups (Figure 4b, all adjusted *p* > 0.05). Similarly, while post‐injury pH was lower in “injured” animals (Figure 4c, *p* = 0.005) there were no statistically significant differences between “single” and “split” ventilated groups (all adjusted *p* > 0.05). Therefore, ability to achieve pH and PaCO_2_ targets in ARDS did not appear superior with standard ventilation compared to “split” ventilation.

#### 3.2.2 BALF sampling

**FIGURE 5:**
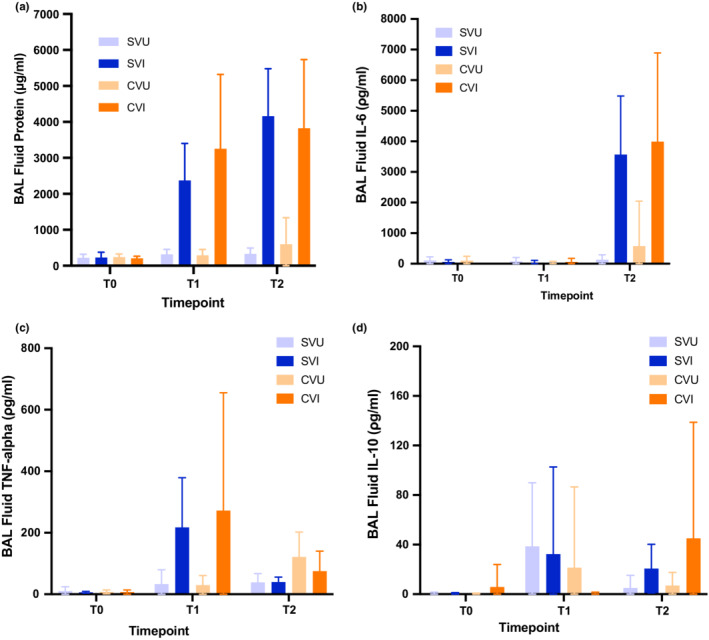
Immunological markers of lung injury. Panel (a) demonstrates BALF protein concentrations in both “split” ventilation and standard ventilation groups of injured and uninjured animals. BALF protein levels were low in all groups at T0 (pre‐injury). Significant increases in BALF protein from baseline were observed in both “split” and standard ventilation groups of injured animals at T1 and T2 (*p* = 0.001). There was no significant difference between “split” and standard ventilation groups. Panel (b) displays bronchoalveolar lavage fluid (BALF) levels of interleukin 6 (IL‐6) in both “split” ventilated and conventionally ventilated injured and uninjured animals. IL‐6 values were low in all groups at T0 (pre‐injury). Levels remain low immediately after injury at T1 in all groups. At T2, following 6 h of ventilation, significant increases in IL‐6 levels were noted in both “split” ventilated injured animals and the standard ventilation group of injured animals compared to uninjured control groups (*p* = 0.006). There was no significant difference between animals who received “split” ventilation and standard ventilation in both the uninjured and injured groups at T2. Panel (c) displays bronchoalveolar lavage fluid (BALF) levels of tumor necrosis factor‐alpha (TNFα) in both the “split” ventilated and standard ventilation groups of injured and uninjured animals. There was no significant difference in BALF TNFα levels between animals who received “split” and standard ventilation in both the uninjured and injured groups at any timepoint. Panel (d) displays bronchoalveolar lavage fluid (BALF) levels of interleukin 10 (IL‐10) in both “split” and standard ventilation groups of injured and uninjured animals. There was no significant difference in BALF IL‐10 levels between animals who received “split” and standard ventilation in both the uninjured and injured groups at any timepoint. SVU = Single (“Standard”) Ventilator Uninjured (*n* = 5); SVI = Single (“Standard”) Ventilator Injured (*n* = 5); CVU = Combi‐Ventilate (“Split”) Uninjured (*n* = 10); CVI = Combi‐Ventilate (“Split”) Injured (*n* = 10).

Because permeability alterations are obvious and severe during ventilator induced oedema (Ricard et al., 2003), BALF levels of protein were quantified across groups (Figure 5a). Post‐injury, BALF protein levels were significantly higher in injured versus uninjured animals (*p* = 0.001). However, there were no significant differences in BALF protein levels between “single” and “split” ventilated animals (all adjusted *p* > 0.05).

Similarly, because BALF levels of IL‐6 are early biomarkers of lung injury (Stüber et al., 2002; Tremblay et al., 1997; Veldhuizen et al., 2001) and predictive of morbidity/mortality in ARDS (Frank et al., 2006; Parsons et al., 2005; Ranieri et al., 1999; Remick et al., 2005), BALF IL‐6 levels were compared across groups (Figure 5b). Baseline BALF IL‐6 levels were low in all groups but significantly increased at 6 h in injured animals (*p* = 0.006). However, there was no significant difference in BALF IL‐6 levels between the “split” and “single” ventilated animals (all adjusted *p*‐values >0.05), suggesting no evidence of additional injury stimulus from “split” ventilation. BALF levels of tumor necrosis factor‐alpha (TNFα) (Figure 5c) and IL‐10 (Figure 5d) displayed similar patterns, with no evidence of additional inflammation in animals undergoing ‘split’ ventilation.

#### 3.2.3 Wet to dry ratios

Wet to dry ratios were used as an index of pulmonary oedema, assuming that if split ventilation produced additional injury it would be associated with higher wet to dry ratios. Mean wet to dry ratios were significantly higher in injured animals (*p* < 0.001) but not in “split” ventilated animals—indeed post hoc testing revealed significantly higher wet to dry ratios in “single” vs “split” ventilated injured animals (*p* = 0.044).

#### 3.2.4 Usability testing

*“S*plit” ventilation systems must be sufficiently intuitive and simple that they can be assembled and used easily. In a convenience sample of critical care staff with minimal training, 17 out of 20 users (85%) successfully assembled the “split” ventilation system. All 20 users (100%) successfully set “parent” ventilator and “split” ventilation module parameters to ventilate test lungs with prescribed tidal volumes. In a simulation of abruptly altered compliance in one test lung, 18 out of 20 users (90%) correctly recognized alarms and responded appropriately. High rates of successful completion of these tasks are consistent with high usability of the “split” ventilation system.

## 4 DISCUSSION

In a porcine model of ARDS, adherence to modified “lung protective” ventilation targets was feasible during “split” ventilation without any appreciable deterioration in markers of pulmonary mechanics, gas exchange or biomarkers of lung injury over a period of approximately 6 hours when compared to standard ventilation. In benchtop testing, “split” ventilation could be used to independently ventilate two test lungs in simulations of clinically significant changes in resistance and compliance. Further, naïve critical care staff could assemble and use a “split” ventilation system in clinical simulations.

These results add significantly to the existing literature. Prior animal studies (Paladino et al., 2008; Stiers et al., 2020) or case series (Beitler et al., 2020; Levin et al., 2020; Smith & Brown, 2009) were all limited by absence of appropriate control groups. This is important because most discussion about “split” ventilation assumes inferiority to standard ventilation (Cook, 2020; Mancebo et al., 2020; The Society of Critical Care Medicine (SCCM) AAfRCA, American Society of Anesthesiologists (ASA), Anesthesia Patient Safety Foundation (APSF), American Association of Critical‐Care Nurses (AACN), and American College of Chest Physicians (CHEST), 2020). Our data therefore significantly inform the question of the safety of “split” ventilation using direct comparison to standard ventilation. Animals in our experiment did not appear to experience additional ventilator‐induced injury when undergoing “split” ventilation. This is consistent with our observation that modified lung protective ventilation was feasible during “split” ventilation and partial individualisation of ventilation achievable. These data should partly reassure those who believe “split” ventilation necessarily represents a lower standard of care (Cook, 2020; Mancebo et al., 2020; The Society of Critical Care Medicine (SCCM) AAfRCA, American Society of Anesthesiologists (ASA), Anesthesia Patient Safety Foundation (APSF), American Association of Critical‐Care Nurses (AACN), and American College of Chest Physicians (CHEST), 2020). Further, it may reframe the ethical “dilemma” posed by choosing between “split” ventilation for two patients or standard ventilation for one patient in surge crises (Cook, 2020; Laffey et al., 2020).

Our benchtop testing demonstrated independent ventilation of two test lungs using a split ventilation system, with the “parent” ventilator set in a pressure‐controlled mode of ventilation. This was true even under conditions of significant variation in compliance or resistance in a single test lung. This is consistent with other bench studies which use variable flow restrictors to “share” ventilation between test lungs with the “parent” ventilator set in a pressure control mode of ventilation (Bishawi et al., 2020; Clarke et al., 2020; Han et al., 2020; Srinivasan et al., 2020). Setting the “parent” ventilator in a pressure‐controlled, rather than a volume‐controlled mode, of ventilation overcomes the potential problem of injurious ventilation being delivered to a single patient in a “split” system when resistance increases or compliance decreases in another patient within the system (Angulo et al., 2020; Branson et al., 2012). This independence of ventilation was observed even under extreme conditions such as disconnection of a single test lung. The independence of ventilation observed in benchtop testing probably partly explains the lack of evidence of superiority of standard ventilation compared to “split” ventilation in our in vivo experiments—“split” ventilation was sufficiently individualized to avoid large injurious effects. While the in vivo experiments themselves provide an index of the feasibility of such systems, the usability tests give further confidence that assembly and use of such systems is intuitive for relevant staff with minimal specific training.

What is the utility of a “split” ventilation system that cannot be assembled with readily available equipment? Several authors have argued that if “split” ventilation has a role, it is as a temporizing measure to allow more standard ventilators to arrive from elsewhere (Mancebo et al., 2020; Paladino et al., 2008). Indeed, it would be too costly to provide all hospitals with enough standard ventilators to meet needs during a “surge” in demand for mechanical ventilation. If a “split” ventilation system capable of individualized lung protective ventilation could be produced cheaply and held at individual sites, this could allow “split” ventilation to act as a crucial temporizing measure at sites experiencing “surges”. A central repository of “spare” ventilators could then supply sites experiencing surge with additional ventilators, allowing de‐escalation from “split” ventilation to standard ventilation. We believe that “split” ventilation devices could be developed sufficiently for such a role. As some authors have pointed out, it must be demonstrated that “split” systems offer advantages over less complex alternatives, particularly manual ventilation (Mancebo et al., 2020). Manual ventilation, while simpler to deliver, has the disadvantage of reduced control of tidal volumes and applied inspiratory pressures (Khoury et al., 2015), potentially risking iatrogenic lung injury in ARDS (Acute Respiratory Distress Syndrome Network et al., 2000). We would argue that the demonstration that lung protective ventilation can be consistently achieved with “split” ventilation systems therefore represents a significant advantage over manual ventilation.

While we report the technical feasibility of ventilating two patients with a single ventilator, there are many areas of caution to consider. We have attempted to address one of the technical challenges of split ventilation, namely individualized tidal volume during conditions of changing lung compliance in one or both ventilated subjects. Although our system was able to individualize tidal volume, we were unable to individualize respiratory rate or fraction of inspired oxygen and we did not individualize positive end‐expiratory pressure. Therefore, close matching of ventilatory settings—such as minute ventilation, positive end‐expiratory pressure and fraction of inspired oxygen—to patient characteristics such as pulmonary mechanics (static compliance, resistance); oxygen consumption and carbon dioxide production; acid–base balance; and haemodynamics—remains necessary in our system to optimize the chance of survival in these severely ill patients. As spontaneous respiratory effort from either patient must be avoided during split ventilation, continuous neuromuscular blockade or deep sedation is required to eliminate the risk of detrimental patient–patient or patient–ventilator interactions. Whether or not the benefit of providing support to one additional patient outweighs the harms suffered by the two patients receiving split ventilation is an impossible question to answer at this point given the lack of evidence and experience.

The role for split ventilation is clearly one of rescue only, an attempt to save those facing certain death, by freeing mechanical ventilators to support those in respiratory failure who would die without them. Unfortunately, given the ongoing pandemic and likely future pandemics with inadequate and unequal distribution of healthcare resources, a forced experience with split ventilation is possible.

### 4.1 Limitations

Our study has several limitations. Because data were not available for similar experiments, power analysis could not be performed. Instead, an optimal sample size was selected using the resource equation method. While this method balances the risk of missing biologically significant effects against the ethical imperative to avoid unnecessary replication, it assumes larger effect sizes and therefore the study likely lacks sufficient power to exclude smaller between group differences. In particular, the data should not be interpreted as demonstrating noninferiority at the level of smaller but potentially clinically relevant effects, since a hypothesis of non‐inferiority was not tested. Due to resource constraints, we were unable to include a comparison of “split” and manual ventilation, a relevant control. Usability tests were based on convenience sampling—more testing will be necessary to ensure the results are generalisable. Furthermore, although our animal model demonstrates feasibility of “split” ventilation as employed by our study team, we cannot exclude operator dependent effects and further testing is warranted. Of course, the results may not be generalisable to other “split” ventilation systems. That said, it seems likely that our in vivo results may be reasonably generalized to other split ventilation systems that operate using flow regulating valves in parallel circuits with the “parent” ventilator set in a “pressure control” mode of ventilation (since our “bench” testing results are concordant with other studies of such systems).

## 5 CONCLUSIONS

We have demonstrated feasibility of “split” ventilation in a porcine model of ARDS and did not find strong evidence of superiority of standard ventilation within the model across a range of measures of pulmonary mechanics, gas exchange, and markers of lung injury. This is an important step in the exploration of a role for “split” ventilation clinically. These data support a rationale for further investigating and developing “split” ventilation systems for use as a temporizing forced rescue measure in clinical practice.

## AUTHOR CONTRIBUTIONS

P.G., J.C., S.M. and G.C. conceptualized the study and designed the experiments. P.G., J.C., G.H., A.K., H.M., K.D., N.Mc.E., and A.D. performed experimental procedures and collected data. P.G., J.C., S.M. and G.C. prepared and analyzed the data. P.G., J.C., S.M., I.M.L, M.P., J.L., and G.C. prepared drafts of the paper. All authors contributed significantly to the revision of the paper for intellectual content. All authors have reviewed the final draft.

## FUNDING INFORMATION

This work was supported with a financial grant (€9,000) from Enterprise Ireland, Dublin, Ireland. Gerard F. Curley is supported by a Health Research Board (Ireland) Emerging Clinician Scientist Award (ESCA ESCA‐2020‐009).

## CONFLICT OF INTEREST

The author declare no competing interests.

## Supporting information


Appendix S1
