# Supplementary material for: Use of a novel “Split” ventilation system in bench and porcine modeling of acute respiratory distress syndrome
Source: Physiol Rep. 2022 Sep 9;10(17):e15452. doi: 10.14814/phy2.15452 (PMC9461348; doi:10.14814/phy2.15452)
Supplement: Supplementary file 1 — Appendix S1 [file PHY2-10-e15452-s001.docx]

**Title: “Split” ventilation in bench and porcine models of Acute Respiratory Distress Syndrome: Online Digital Supplement.**

**Authors:**

*Pierce Geoghegan^1^ (MB BCh BAO), Jennifer Clarke^1^ (MB BCh BAO), Grace Hogan^1^(MSc), Aoife Keogh^1^(MSc), Hannah Marsh^2^(MB BCh BAO), Karen Donnelly^1^ (MB BCh BAO), Natalie McEvoy^1^ (MSc), Aoife Doolan^3^(MB BCh BAO), Stephen F Madden^4^(PhD), Ignacio Martin-Loeches^1^(PhD), Michael Power^2^ (MB BCh BAO), John G Laffey^5^(PhD), Gerard F Curley^1^* (PhD).

1. Department of Anaesthesia and Critical Care, Royal College of Surgeons in Ireland, Dublin, Ireland.
2. Beaumont Hospital, Dublin, Ireland.
3. Tallaght Hospital, Dublin, Ireland.
4. Data Science Centre, Royal College of Surgeons in Ireland, Dublin, Ireland.
5. Department of Intensive Care, St. James Hospital, Dublin, Ireland.
6. Department of Anaesthesia and Critical Care, Galway University Hospital, Galway Ireland.

**Institution:** Department of Anaesthesia and Critical Care, Beaumont Hospital, Ireland

**Corresponding author:** Prof G Curley.

**Corresponding author contact details:** Department of Anaesthesia and Critical Care, Royal College of Surgeons in Ireland, Dublin, Ireland (email: gercurley@rcsi.ie).

**Supplementary Methods**

*S1.1 “Parent” ventilator settings for benchtop testing and benchtop testing with Draeger Models of Ventilator*

The “parent” Servo-I ventilator was set in a pressure control mode with the following settings; respiratory rate 20, FiO_2_ 0.4, positive end-expiratory pressure (PEEP) 5-10cm H_2_O, pressure control above PEEP 25-30cmH_2_0. We performed similar benchtop testing with two other common models of ventilator with similar settings in the relevant modes of ventilation (V800, Dräger medical, Lübeck, Germany and Evita 4; Dräger medical, Lübeck, Germany). The system assembly is as described in the main manuscript and illustrated there in Figure 1. For Draeger models, an additional connection was necessary between the inflow and outflow circuitry (on the ventilator side of the one way valves) to prevent ventilator pressure differential pressure alarm activation (shown in figure E1 below).

*S1.2 Animal Preparation, Treatment Groups, and mechanical ventilation settings and targets*

Thirty local community-bred female Landrace pigs (23 to 56kg, mean 40kg +/- 10kg ) were intramuscularly premedicated with midazolam (1 mg kg^-1^) and ketamine (10 mg kg^-1^). A 24 gauge cannula was inserted into the ear vein and intravenous anaesthesia induced with a bolus of 2mg kg^-1^ propofol and 10mcg kg^-1^ of fentanyl. Anaesthesia was maintained with propofol (2-4mg kg^-1^ hr^-1^), midazolam (1.25mg kg^-1^ hr^-1^) and fentanyl (5mcg kg^-1^ hr^-1^); paralysis was achieved with vecuronium (bolus of 0.6mg kg^-1^, followed by an infusion of 0.3mg kg^-1^ hr^-1^). The anaesthesia regimen included a strong opioid (fentanyl) for analgesia. After induction of general anaesthesia, and application of lignocaine spray (1% w/v) to the larynx, direct laryngoscopy was performed, and the pigs were orally intubated (7.0mm internal diameter cuffed endotracheal tube). Prior to the administration of neuromuscular blocking agents, adequate depth of anaesthesia was confirmed by non-response to a compressive toe pinch. Following the administration of neuromuscular blocking agents, depth of anaesthesia was monitored by observing changes in heart rate and blood pressure in response to stimulating study procedures. Mechanical ventilation was commenced with the relevant ventilator, using initial settings of tidal volume (6-8ml kg^-1^ actual body weight), respiratory rate (30 breaths per minute), PEEP (5-10cmH_2_0), and FiO_2_ of 1.0. A recruitment manoeuvre was performed in all subjects comprising an end inspiratory hold manoeuvre at 25cmH_2_O for 30 seconds in all subjects prior to arterial blood gas sampling.

A 20G arterial cannula was inserted into the femoral artery under ultrasound guidance using a Seldinger technique for the purposes of continuous arterial blood pressure monitoring and blood sampling. Similarly, a triple lumen central venous catheter was inserted into the femoral vein under ultrasound guidance to facilitate infusion of medications and fluids. Intravenous compound sodium lactate was infused at a rate of 5ml kg^-1^ hr^-1^ to a maximum of 30ml kg^-1^. Infusions of noradrenaline (0.1-0.5mcg kg^-1^ min^-1^) and vasopressin (0-2.4 IU per hour) were used to maintain mean arterial pressure > 65mmHg during general anaesthesia. Temperature was maintained at 37-39 degrees Celsius using electronically controlled heating pads.

Replicates were assigned to one of four main treatment groups – “single” ventilated uninjured, “single” ventilated injured, “split” ventilated uninjured and “split” ventilated injured. Block allocation of replicates was performed with a block size of 4, primarily to control for learning effects. There were 5 replicates per group. The Combi-Ventilate system necessitated ventilation of two animals simultaneously and so each pair of animals was a single replicate. In total there were 30 pigs (5 “single” ventilated uninjured, 5 “single” ventilated injured, 10 “split” ventilated uninjured, and 10 “split” ventilated injured).

Two pigs were ventilated on each study day, either two “single” ventilated or two “split” ventilated animals, to prevent confounding by resource depletion on “split” ventilation days. Pigs in the “single” ventilated groups were ventilated using a conventional ventilator (Evita 4; Dräger medical, Lübeck, Germany). Pigs in the “split” ventilated groups were ventilated using the Combi-Ventilate system with the Servo-I (Maquet) acting as the “parent” ventilator.

Animals in all groups were ventilated in a “volume control” mode of ventilation. Initial tidal volume was set at 6-8ml kg^-1^ for all pigs with a respiratory rate of 30 breaths per minute and PEEP was set at 5-10cmH_2_O. For pigs ventilated with the Combi-Ventilate device, PEEP, respiratory rate and FiO_2_ were set on the “parent” ventilator and tidal volume was set on the individual animal’s Combi-Ventilate module. These parameters could be adjusted to achieve a plateau pressure ≤30cmH_2_0, with a tidal volume of 6-8ml kg^-1^ and an arterial blood pH>7.15. If the pH target could not be achieved without breaching the tidal volume or plateau pressure goals, we permitted intravenous administration of sodium bicarbonate 8.4% w/v up to 4ml kg^-1^ to achieve the pH goal. In “single” ventilated animals, PEEP was set according to the ARDSnet “low” PEEP protocol (based on FiO_2_ to achieve oxygenation targets of PaO_2_>60mmHg or SaO_2_>88%). In “split” ventilated animals, it was not possible to individualise PEEP and PEEP was titrated to the lowest PEEP which allowed oxygenation targets to be reached in both animals.

Animals were euthanised upon completion of the experiment, or if humane endpoints were reached, by intravenous injection of Somulose (Secobarbital Sodium 400 mg/Cinchocaine Hydrochloride 25 mg), 0.1ml kg^-1^ body weight. Humane endpoints were never reached during the experiments.

*Lung Injury Protocol:*

Lung injury was accomplished by endobronchial administration of acid. HCl 0.05N, pH 1.41, was prepared and instilled (8 ml kg^-1^ body weight) at the right cranial lobe bronchus, the right main bronchus and the left main bronchus, in the ratio of 1:2:3 over 3 min by means of a flexible bronchoscope (Ambu®ascope™). We instilled the acid directly after intubation and allowed 60 minutes post instillation for lung injury to become established. In uninjured animals, bronchoscopy was performed at identical timepoints, but without any instillation of acid or vehicle.

S1.3 *Respiratory Mechanics and Gas exchange*

Total respiratory system compliance was calculated as tidal volume/(plateau pressure-PEEP). For “single” ventilated animals, plateau pressure was measured during an end inspiratory hold and PEEP during an end expiratory hold. For “split” ventilated animals, plateau pressure was measured by the Combi-Ventilate spirometer during an end inspiratory hold on the “parent” ventilator. Similarly, PEEP was measured by the Combi-Ventilate spirometer system during an end expiratory hold manoeuvre on the “parent” ventilator (Combi-Ventilate measurements of PEEP and plateau pressure were validated separately).

Gas exchange was determined hourly by arterial blood sampling (ABL 5; Radiometer; Copenhagen, Denmark). SaO2 was determined by co-oximetry. PF ratios were calculated as PaO_2_/FiO_2_.

S1.4 *Bronchoalveolar Lavage Fluid (BALF) sampling and processing*

Bronchoalveolar lavage fluid (BALF) sampling was performed at three discrete timepoints during the experiment. The first BALF sample was obtained immediately following intubation. The second BALF sample was obtained one hour post instillation of acid or at an identical timepoint in uninjured animals. The third sample was taken after 6 hours of post injury mechanical ventilation prior to euthanasia. Samples were obtained from the right cranial lobe bronchus. 20mls of 0.9% NaCl was instilled and immediately aspirated. Samples were stored on ice and processed within 1 hour of sampling.

Samples were centrifuged at 1500rpm for 10 minutes at 4 degrees Celsius. Supernatant was centrifuged at 1200rpm for a further 8 minutes at 4 degrees Celsius. Supernatant was stored in 1ml aliquots at minus 80 degrees Celsius.

Total protein levels in BAL were quantified using the Pierce^™^ BCA protein assay kit from Thermo Scientific. In brief, 10μl standards and samples were added in duplicate wells to a 96-well plate. Subsequently, 200μl BCA reagent (50 parts reagent A : 1 part reagent B) was added to each well and incubated in the dark for 30 minutes at 37^o^C. The optical density was measured spectrophotometrically at 544nm and total protein levels were quantified using the equation of the line: y=mX+c.

ELISA kits from R&D systems were used to analyse the levels of of IL-6 (DY686), IL-10 (DY693B) and TNFa (DY690B) in BALF. Levels were examined in samples taken immediately before injury, 20 minutes post-injury and 6 hours later (In brief, 96-well plated were coated in 100μl capture antibody overnight. The following day, the plate was washed and blocked with 100μl reagent diluent. The plate was washed again and 100μl standards and samples were added and incubated for 2 hours. The plate was washed again and 100μl detection antibody was added to the wells and incubated for a further 2 hours. Subsequently, the plate was washed and 100μl streptavidin-HRP was added to each well and incubated for 20 minutes in the dark. The plate was washed and 100μl of substrate solution was added and incubated for 20 minutes in the dark. Finally, 50μl stop solution was added to stop the reaction and complete the ELISA. The optical density was measured spectrophotometrically at 450nm and 590nm and cytokine levels were quantified using MyCurveFit software.)

**Supplementary Results**

Table S1.

*Maximum Discrepancy in tidal volumes achieved at varying matched compliance of two test lungs*

| **Driving Pressure**  **(cmH_2_O)** | **Compliance - Test Lungs** | **Tidal Volume Parent Ventilator** | **Tidal Volume Test Lung A** | **Tidal Volume Test Lung B** |
| --- | --- | --- | --- | --- |
| 20 | 80 | 1280 | 970 | 220 |
| 20 | 50 | 1200 | 870 | 230 |
| 20 | 20 | 750 | 560 | 240 |

| Test_____ | Parent Measured PEEP | Parent Measured Peak Pressure | C Lung A | C Lung B | Resistance Lung A | Resistance Lung B | PEEP Lung A | Peak Pressure Lung A | Tidal Volume Lung A | PEEP Lung B | Peak Pressure Lung B | Tidal Volume Lung B | % Decrease Tidal Volume in Partner Lung |
| --- | --- | --- | --- | --- | --- | --- | --- | --- | --- | --- | --- | --- | --- |
| A | 9.9 | 39 | 100 | 100 | 5 | 5 | 15 | 20 | 413 | 13 | 19 | 399 |  |
|  | 9.8 | 39 | 100 | 80 | 5 | 5 | 14 | 20 | 409 | 13 | 20 | 403 | 0.97 |
|  | 9.8 | 39 | 100 | 60 | 5 | 5 | 14 | 20 | 410 | 13 | 19 | 396 | 0.73 |
|  | 9.7 | 39 | 100 | 40 | 5 | 5 | 14 | 20 | 410 | 13 | 23 | 383 | 0.73 |
|  | 10 | 39 | 100 | 30 | 5 | 5 | 14 | 20 | 407 | 12 | 26 | 368 | 1.45 |
|  | 9.9 | 39 | 80 | 80 | 5 | 5 | 14 | 20 | 408 | 13 | 18 | 402 |  |
|  | 9.8 | 39 | 80 | 60 | 5 | 5 | 14 | 19 | 405 | 13 | 21 | 397 | 0.74 |
|  | 10 | 39 | 80 | 40 | 5 | 5 | 14 | 20 | 404 | 12 | 21 | 385 | 0.98 |
|  | 10 | 39 | 80 | 30 | 5 | 5 | 14 | 20 | 404 | 12 | 26 | 365 | 0.98 |
|  | 9.9 | 39 | 60 | 60 | 5 | 5 | 14 | 21 | 402 | 13 | 21 | 400 |  |
|  | 10 | 39 | 60 | 40 | 5 | 5 | 14 | 21 | 403 | 12 | 23 | 389 | -0.25 |
|  | 10 | 39 | 60 | 30 | 5 | 5 | 14 | 21 | 403 | 12 | 26 | 363 | -0.25 |
|  | 10 | 39 | 40 | 40 | 5 | 5 | 13 | 24 | 399 | 12 | 24 | 401 |  |
|  | 10 | 39 | 40 | 30 | 5 | 5 | 13 | 24 | 400 | 12 | 26 | 378 | -0.25 |
|  | 10 | 39 | 30 | 30 | 5 | 5 | 13 | 26 | 393 | 12 | 27 | 399 |  |
|  | 10 | 39 | 30 | 80 | 5 | 5 | 13 | 26 | 390 | 14 | 21 | 440 | 0.76 |
| B | 9.9 | 39 | 80 | 80 | 5 | 5 | 14 | 18 | 394 | 14 | 20 | 399 |  |
|  | 10 | 39 | 80 | 80 | 5 | 20 | 14 | 20 | 399 | 14 | 23 | 360 | -1.27 |
|  | 9.7 | 39 | 50 | 50 | 5 | 5 | 14 | 22 | 400 | 13 | 22 | 405 |  |
|  | 10 | 39 | 50 | 50 | 5 | 20 | 14 | 22 | 401 | 13 | 25 | 369 | -0.25 |
|  | 10 | 39 | 30 | 30 | 5 | 5 | 13 | 26 | 390 | 12 | 27 | 387 |  |
|  | 10 | 39 | 30 | 30 | 5 | 20 | 13 | 26 | 391 | 12 | 28 | 359 | -0.26 |
| C | 10 | 39 | 30 | 30 | 5 | 5 | 13 | 26 | 388 | 12 | 28 | 397 |  |
|  | 9.4 | 38 | 30 | 30 | 5 | *a* | 13 | 26 | 380 | - | - | - | 2.06 |
|  | 10 | 39 | 30 | 30 | 5 | 5 | 13 | 26 | 390 | 12 | 28 | 398 |  |
|  | 9.8 | 38 | 30 | 30 | 5 | *b* | 13 | 26 | 388 | - | - | - | 0.51 |
| D | 10 | 39 | 30 | 30 | 5 | 5 | 13 | 26 | 391 | 12 | 27 | 399 |  |
|  | 10 | 39 | 30 | 30 | 5 | *-* | 13 | 26 | 394 | - | - | - | -0.77 |

**Table S2.** Demonstration of ventilatory independence of two test lungs under conditions of changing compliance or resistance in a single test lung with the V800 ventilator (Dräger medical, Lübeck, Germany) acting as the parent ventilator. Panel A displays the effect of changing one test lung’s (lung B) compliance in different conditions of initially matched compliance. Panel B represents the effects of increased resistance under different conditions of matched compliance. Panel C represents the effects of a disconnect from one test lung under conditions of matched compliance where “a” represents a disconnect at the ET tube and “b” represents a disconnect distal to the Y piece. Panel D represents the effect of an occlusion at test lung B under conditions of matched compliance. C=compliance.

| Test | Parent Measured PEEP | Parent Measured Peak Pressure | C Lung A | C Lung B | Resistance Lung A | Resistance Lung B | PEEP Lung A | Peak Pressure Lung A | Tidal Volume Lung A | PEEP Lung B | Peak Pressure Lung B | Tidal Volume Lung B | % Decrease Tidal Volume in Partner Lung |
| --- | --- | --- | --- | --- | --- | --- | --- | --- | --- | --- | --- | --- | --- |
| A | 10 | 40 | 100 | 100 | 5 | 5 | 15 | 20 | 395 | 14 | 20 | 399 |  |
|  | 10 | 40 | 100 | 80 | 5 | 5 | 15 | 19 | 396 | 14 | 20 | 394 | -0.25 |
|  | 10 | 40 | 100 | 60 | 5 | 5 | 15 | 20 | 395 | 13 | 21 | 393 | 0.00 |
|  | 10 | 40 | 100 | 40 | 5 | 5 | 15 | 20 | 394 | 13 | 23 | 376 | 0.25 |
|  | 10 | 40 | 100 | 30 | 5 | 5 | 15 | 20 | 396 | 12 | 26 | 363 | -0.25 |
|  | 10 | 40 | 80 | 80 | 5 | 5 | 14 | 20 | 393 | 14 | 20 | 397 |  |
|  | 10 | 40 | 80 | 60 | 5 | 5 | 15 | 20 | 395 | 13 | 21 | 395 | -0.51 |
|  | 10 | 40 | 80 | 40 | 5 | 5 | 15 | 20 | 393 | 13 | 24 | 379 | 0.00 |
|  | 10 | 40 | 80 | 30 | 5 | 5 | 15 | 20 | 398 | 13 | 26 | 357 | -1.27 |
|  | 10 | 40 | 60 | 60 | 5 | 5 | 14 | 21 | 395 | 13 | 21 | 401 |  |
|  | 10 | 40 | 60 | 40 | 5 | 5 | 14 | 21 | 395 | 13 | 24 | 384 | 0.00 |
|  | 10 | 40 | 60 | 30 | 5 | 5 | 14 | 21 | 396 | 12 | 26 | 362 | -0.25 |
|  | 10 | 40 | 40 | 40 | 5 | 5 | 14 | 24 | 394 | 13 | 24 | 404 |  |
|  | 10 | 40 | 40 | 30 | 5 | 5 | 14 | 24 | 393 | 13 | 27 | 386 | 0.25 |
|  | 10 | 40 | 30 | 30 | 5 | 5 | 13 | 27 | 386 | 12 | 27 | 389 |  |
|  | 10 | 40 | 30 | 80 | 5 | 5 | 13 | 27 | 390 | 14 | 21 | 429 | -1.04 |
| B | 10 | 40 | 80 | 80 | 5 | 5 | 14 | 20 | 394 | 14 | 20 | 398 |  |
|  | 10 | 40 | 80 | 80 | 5 | 20 | 14 | 20 | 393 | 14 | 23 | 368 | 0.25 |
|  | 10 | 40 | 50 | 50 | 5 | 5 | 14 | 22 | 394 | 13 | 22 | 394 |  |
|  | 10 | 40 | 50 | 50 | 5 | 20 | 14 | 22 | 389 | 13 | 24 | 359 | 1.27 |
|  | 10 | 40 | 30 | 30 | 5 | 5 | 13 | 26 | 387 | 13 | 27 | 394 |  |
|  | 10 | 40 | 30 | 30 | 5 | 20 | 13 | 27 | 388 | 13 | 29 | 358 | -0.26 |
| C | 10 | 40 | 30 | 30 | 5 | 5 | 13 | 26 | 386 | 13 | 27 | 388 |  |
|  | 10 | 39 | 30 | 30 | 5 | *a* | 13 | 26 | 389 | - | - | - | -0.78 |
|  | 10 | 40 | 30 | 30 | 5 | 5 | 13 | 27 | 387 | 13 | 27 | 394 |  |
|  | 10 | 39 | 30 | 30 | 5 | *b* | 13 | 26 | 389 | - | - | - | -0.52 |
| D | 10 | 40 | 30 | 30 | 5 | 5 | 13 | 37 | 387 | 13 | 27 | 394 |  |
|  | 10 | 40 | 30 | 30 | 5 | *-* | 13 | 27 | 390 | - | - | - | -0.78 |

**Table S3.** Demonstration of ventilatory independence of two test lungs under conditions of changing compliance or resistance in a single test lung with the Evita 4 ventilator (Dräger medical, Lübeck, Germany) acting as the parent ventilator. Panel A displays the effect of changing one test lung’s (lung B) compliance in different conditions of initially matched compliance. Panel B represents the effects of increased resistance under different conditions of matched compliance. Panel C represents the effects of a disconnect from one test lung under conditions of matched compliance where “a” represents a disconnect at the ET tube and “b” represents a disconnect distal to the Y piece. Panel D represents the effect of an occlusion at test lung B under conditions of matched compliance. C=Compliance.

| Test | Parent Measured PEEP | Parent Measured Peak Pressure | C Lung A | C Lung B | Resistance Lung A | Resistance Lung B | PEEP Lung A | Peak Pressure Lung A | Tidal Volume Lung A | PEEP Lung B | Peak Pressure Lung B | Tidal Volume Lung B | % Decrease Tidal Volume in Partner Lung |
| --- | --- | --- | --- | --- | --- | --- | --- | --- | --- | --- | --- | --- | --- |
| A | 14 | 20 | 100 | 100 | 5 | 5 | 14 | 20 | 399 | 13 | 19 | 411 |  |
|  | 14 | 20 | 80 | 100 | 5 | 5 | 14 | 20 | 386 | 13 | 19 | 408 | 0.73 |
|  | 14 | 21 | 60 | 100 | 5 | 5 | 14 | 21 | 385 | 13 | 19 | 411 | 0.00 |
|  | 13 | 26 | 30 | 100 | 5 | 5 | 13 | 26 | 351 | 13 | 19 | 407 | 0.97 |
|  | 13 | 23 | 40 | 100 | 5 | 5 | 13 | 24 | 372 | 13 | 19 | 408 | 0.73 |
|  | 14 | 20 | 80 | 80 | 5 | 5 | 14 | 20 | 390 | 13 | 20 | 401 |  |
|  | 13 | 21 | 60 | 80 | 5 | 5 | 14 | 21 | 382 | 13 | 20 | 401 | 0.00 |
|  | 13 | 23 | 40 | 80 | 5 | 5 | 12 | 22 | 390 | 13 | 20 | 405 | -1.00 |
|  | 13 | 26 | 30 | 80 | 5 | 5 | 12 | 25 | 371 | 13 | 20 | 401 | 0.00 |
|  | 13 | 21 | 60 | 60 | 5 | 5 | 13 | 20 | 397 | 13 | 21 | 397 |  |
|  | 13 | 23 | 40 | 60 | 5 | 5 | 12 | 23 | 385 | 13 | 21 | 396 | 0.25 |
|  | 13 | 26 | 30 | 60 | 5 | 5 | 12 | 25 | 360 | 13 | 21 | 397 | 0.00 |
|  | 13 | 23 | 40 | 40 | 5 | 5 | 12 | 22 | 386 | 12 | 24 | 392 |  |
|  | 13 | 23 | 30 | 40 | 5 | 5 | 12 | 25 | 365 | 12 | 19 | 390 | 0.51 |
|  | 12 | 26 | 30 | 30 | 5 | 5 | 12 | 25 | 387 | 12 | 27 | 388 |  |
|  | 13 | 26 | 80 | 30 | 5 | 5 | 13 | 19 | 424 | 12 | 27 | 397 | -2.32 |
| B | 16 | 21 | 80 | 80 | 5 | 5 | 13 | 17 | 405 | 12 | 18 | 413 |  |
|  | 14 | 23 | 80 | 80 | 5 | 20 | 13 | 19 | 404 | 13 | 22 | 378 | 0.25 |
|  | 13 | 22 | 50 | 50 | 5 | 5 | 12 | 21 | 394 | 12 | 20 | 390 |  |
|  | 13 | 23 | 50 | 50 | 5 | 20 | 12 | 21 | 392 | 12 | 23 | 361 | 0.51 |
|  | 13 | 27 | 30 | 30 | 5 | 5 | 11 | 26 | 401 | 11 | 27 | 399 |  |
|  | 13 | 29 | 30 | 30 | 5 | 20 | 12 | 26 | 402 | 11 | 29 | 381 | -0.25 |
| C | 12 | 27 | 30 | 30 | 5 | 5 | 12 | 26 | 400 | 11 | 27 | 404 |  |
|  | 1 | 28 | 30 | 30 | 5 | *a* | 12 | 27 | 401 | - | - | - | -0.25 |
|  | 13 | 27 | 30 | 30 | 5 | 5 | 12 | 26 | 401 | 11 | 27 | 409 |  |
|  | 1 | 28 | 30 | 30 | 5 | *b* | 12 | 27 | 389 | - | - | - | 2.99 |
| D | 12 | 27 | 30 | 30 | 5 | 5 | 12 | 26 | 408 | 11 | 27 | 406 |  |
|  | 13 | 39 | 30 | 30 | 5 | - | 11 | 24 | 337 | - | - | - | 17.40 |

**Table S4.** Demonstration of ventilatory independence of two test lungs under conditions of changing compliance or resistance in a single test lung with the Servo-I (Maquet, Germany) acting as the parent ventilator with original set up as shown in main text . Panel A displays the effect of changing one test lung’s (lung A) compliance in different conditions of initially matched compliance. Panel B represents the effects of increased resistance under different conditions of matched compliance. Panel C represents the effects of a disconnect from one test lung under conditions of matched compliance where “a” represents a disconnect at the ET tube and “b” represents a disconnect distal to the Y piece. Panel D represents the effect of an occlusion at test lung B under conditions of matched compliance. C=compliance.

| Test | Parent Measured PEEP | Parent Measured Peak Pressure | C Lung A | C Lung B | Resistance Lung A | Resistance Lung B | PEEP Lung A | Peak Pressure Lung A | Tidal Volume Lung A | PEEP Lung B | Peak Pressure Lung B | Tidal Volume Lung B | % Decrease Tidal Volume in Partner Lung |
| --- | --- | --- | --- | --- | --- | --- | --- | --- | --- | --- | --- | --- | --- |
| A | 10 | 40 | 100 | 100 | 5 | 5 | 14 | 17 | 409 | 13 | 19 | 391 |  |
|  | 10 | 40 | 80 | 100 | 5 | 5 | 13 | 20 | 407 | 13 | 18 | 394 | -0.77 |
|  | 10 | 40 | 60 | 100 | 5 | 5 | 13 | 21 | 399 | 13 | 19 | 407 | -4.09 |
|  | 10 | 40 | 40 | 100 | 5 | 5 | 12 | 23 | 384 | 13 | 19 | 401 | -2.56 |
|  | 10 | 40 | 30 | 100 | 5 | 5 | 12 | 25 | 365 | 13 | 19 | 399 | -2.05 |
|  | 10 | 40 | 80 | 80 | 5 | 5 | 13 | 20 | 392 | 12 | 19 | 395 |  |
|  | 10 | 40 | 60 | 80 | 5 | 5 | 13 | 20 | 382 | 12 | 19 | 392 | 0.76 |
|  | 10 | 40 | 40 | 80 | 5 | 5 | 12 | 23 | 367 | 12 | 19 | 406 | -2.78 |
|  | 10 | 40 | 30 | 80 | 5 | 5 | 12 | 25 | 351 | 12 | 19 | 396 | -0.25 |
|  | 10 | 40 | 60 | 60 | 5 | 5 | 13 | 20 | 387 | 12 | 20 | 385 |  |
|  | 10 | 40 | 40 | 60 | 5 | 5 | 12 | 23 | 375 | 12 | 20 | 385 | 0.00 |
|  | 10 | 40 | 30 | 60 | 5 | 5 | 12 | 25 | 353 | 12 | 20 | 389 | -1.04 |
|  | 10 | 40 | 40 | 40 | 5 | 5 | 12 | 23 | 393 | 12 | 23 | 394 |  |
|  | 10 | 40 | 30 | 40 | 5 | 5 | 12 | 25 | 371 | 12 | 23 | 396 | -0.51 |
|  | 10 | 40 | 30 | 30 | 5 | 5 | 12 | 26 | 390 | 11 | 27 | 402 |  |
|  | 10 | 40 | 80 | 30 | 5 | 5 | 13 | 21 | 433 | 11 | 27 | 405 | -0.75 |
| B | 10 | 40 | 80 | 80 | 5 | 5 | 13 | 20 | 418 | 13 | 19 | 395 |  |
|  | 10 | 40 | 80 | 80 | 5 | 20 | 13 | 20 | 406 | 13 | 22 | 358 | 2.87 |
|  | 10 | 40 | 50 | 50 | 5 | 5 | 13 | 22 | 396 | 12 | 22 | 416 |  |
|  | 10 | 40 | 50 | 50 | 5 | 20 | 13 | 22 | 393 | 12 | 23 | 376 | 0.76 |
|  | 10 | 40 | 30 | 30 | 5 | 5 | 12 | 26 | 391 | 11 | 27 | 410 |  |
|  | 10 | 40 | 30 | 30 | 5 | 20 | 12 | 26 | 392 | 12 | 28 | 374 | -0.26 |
| C | 10 | 40 | 30 | 30 | 5 | 5 | 12 | 26 | 384 | 11 | 27 | 411 |  |
|  | 9 | 40 | 30 | 30 | 5 | *a* | 14 | 28 | 356 | - | - | - | 7.29 |
|  | 10 | 40 | 30 | 30 | 5 | 5 | 12 | 27 | 405 | 11 | 27 | 409 |  |
|  | 10 | 40 | 30 | 30 | 5 | *b* | 14 | 28 | 368 | - | - | - | 9.14 |
| D | 10 | 40 | 30 | 30 | 5 | 5 | 12 | 27 | 410 | 11 | 27 | 406 |  |
|  | 10 | 40 | 30 | 30 | 5 | - | 12 | 27 | 410 | - | - | - | 0.00 |

**Table S5.** Demonstration of ventilatory independence of two test lungs under conditions of changing compliance or resistance in a single test lung with the Servo I (Maquet, Germany) acting as the parent ventilator and with the circuit modified to contain a connection between the inflow and outflow ports of the parent ventilator at the ventilator side of the one way flow valves. Panel A displays the effect of changing one test lung’s (lung A) compliance in different conditions of initially matched compliance. Panel B represents the effects of increased resistance under different conditions of matched compliance. Panel C represents the effects of a disconnect from one test lung under conditions of matched compliance where “a” represents a disconnect at the ET tube and “b” represents a disconnect distal to the Y piece. Panel D represents the effect of an occlusion at test lung B under conditions of matched compliance. C=compliance.

**Additional Supplemental Content:**

*Table S6. Components of Combiventilate System*

| **Component Name** | **Brand** | **Quantity** |
| --- | --- | --- |
| End Expiratory Filter | Fisher and Paykel | 4 |
| Adult breathing circuit | Fisher and Paykel | 2 |
| HME Filter | Romsons HME | 2 |
| Adult patient spirometry set 2m | Intersurgical | 2 |
| One way flow valves | Intersurgical | 4 |
| T piece connectors | Intersurgical | 2 |
| Female Female adaptors | Intersurgical | 4 |

*Table S7. Sample data from validation of Combi-Ventilate plateau pressure measurements*

| **Set Compliance Michigan Lung 1** | **Set Compliance Michigan Lung 2** | **Plateau Pressure Combi-Ventilate 1** | **Intra-Pleaural Pressure Michigan Lung 1** | **% Difference Plateau vs Intra-Pleural Pressures 1** | **PEEP Lung 1** | **Tidal Volume Lung 1** | **Plateau Pressure Combi-Ventilate 2** | **Intra-Pleaural Pressure Michigan Lung 2** | **% Difference Plateau vs Intra-Pleural Pressures 2** | **PEEP Lung 2** | **Tidal Volume Lung 2** |
| --- | --- | --- | --- | --- | --- | --- | --- | --- | --- | --- | --- |
| 30 | 30 | 25.5 | 25 | 1.96 | 12 | 409 | 26 | 26 | 0.00 | 12 | 411 |
| 50 | 50 | 20.7 | 20 | 3.38 | 13 | 407 | 20.9 | 19 | 9.09 | 12 | 408 |
| 80 | 80 | 18.4 | 18 | 2.17 | 13 | 400 | 18 | 17 | 5.56 | 13 | 397 |
| 30 | 80 | 22 | 22 | 0.00 | 12 | 396 | 18.9 | 18 | 4.76 | 13 | 393 |

*Figure E1.* displays the modified circuit set up for use with Drager ventilators with an additional connection between the inflow and outflow circuitry (on the ventilator side of the one-way valves) to prevent ventilator pressure differential pressure alarm activation.

**
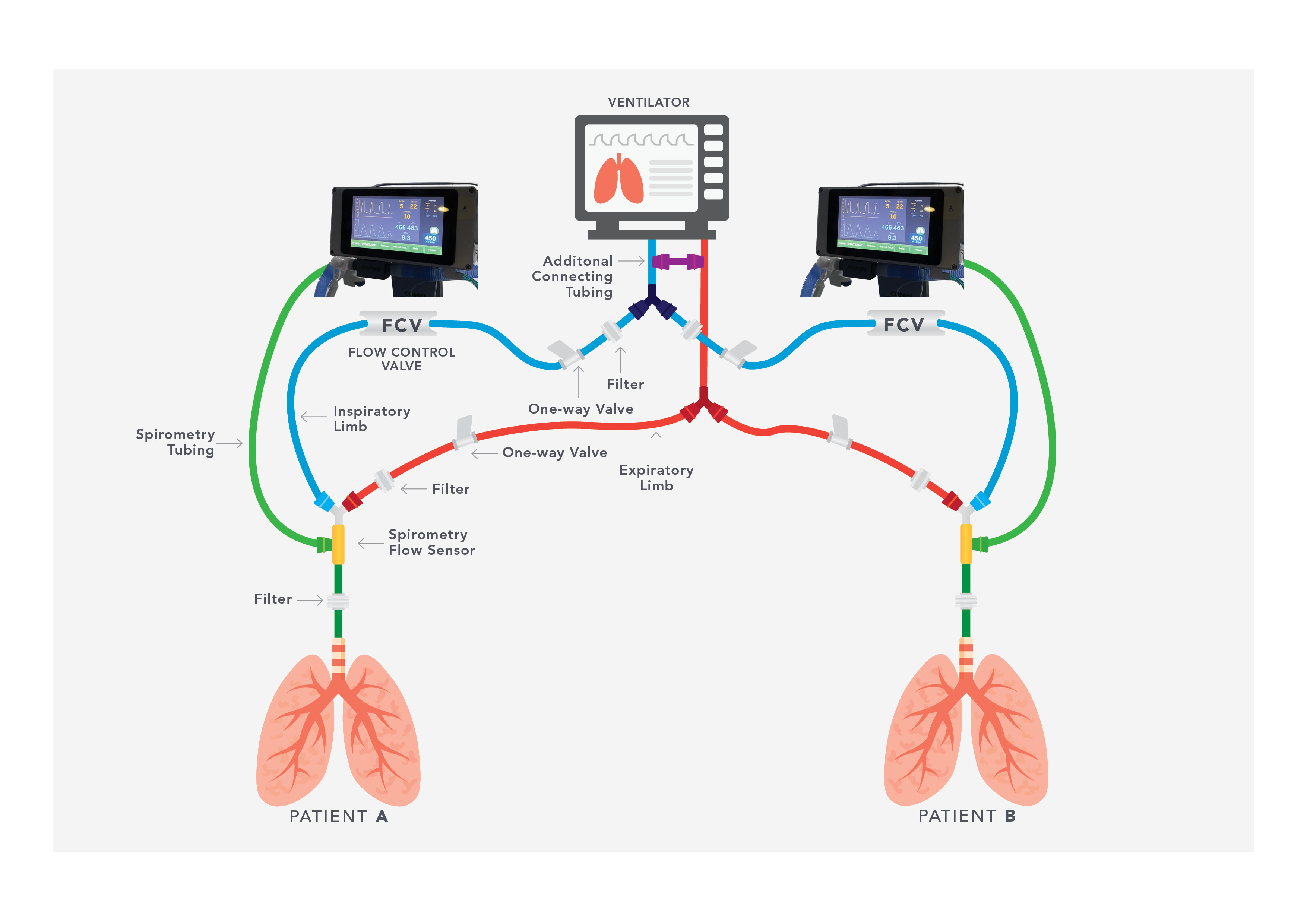
**

**Figure E2. Measurements of wet:dry ratios of post mortem lung tissue samples in “split” and conventionally ventilated injured and uninjured animals.** Mean wet to dry ratios were significantly higher in injured animals (p=<0.001) but not in “split” ventilated animals. Post hoc testing revealed significantly higher wet to dry ratios in “single” vs “split” ventilated injured animals (p=0.044). Differences in treatments were determined using a two-way repeated measures ANOVA. Pairwise comparisons between ventilation types were performed using a t-test and p-values were adjusted using the Benjamini-Hochberg method. SVU=Single Ventilator Uninjured (n=5); SVI=Single Ventilator Injured (n=5); CVU= Combi-Ventilate Uninjured (n=10); CVI= Combi-Ventilate Injured (n=10).


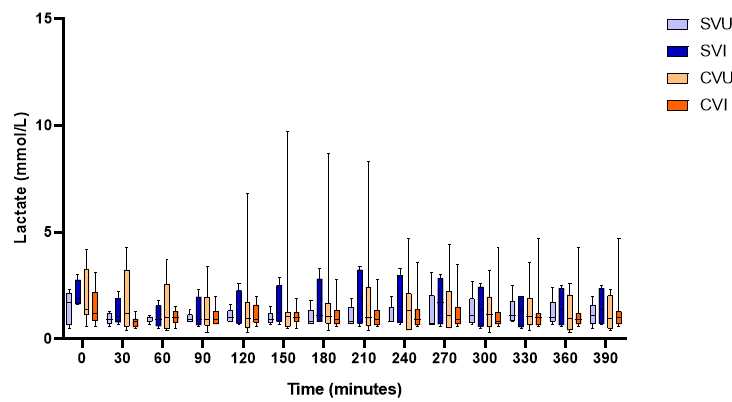


**Figure E3. Serial measurements of arterial lactate in injured and uninjured animals undergoing “conventional” and “split” ventilation.** Data are presented as box and whisker plots. The box indicates the interquartile range and contains a line at the median value. The whiskers denote the range. SVU=Single Ventilator Uninjured (n=5); SVI=Single Ventilator Injured (n=5); CVU= Combi-Ventilate Uninjured (n=10); CVI= Combi-Ventilate Injured (n=10).
